# Increasing capacity for ethnically-based community leaders to engage in policy change: assessing the impact of a train-the-trainer approach

**DOI:** 10.1186/s12889-024-20822-0

**Published:** 2025-03-11

**Authors:** Angel Lomeli, Nicole A. Stadnick, Kelli L. Cain, Paul Watson, William Oswald, Shelia L. Broyles, Marina Ibarra, Borsika Rabin

**Affiliations:** 1 Herbert Wertheim School of Public Health and Human Longevity Sciences, University of California San Diego, La Jolla, CA USA; 2 Department of Obstetrics, Gynecology, and Reproductive Sciences, University of California San Diego Health, La Jolla, CA USA; 3 UC San Diego Altman Clinical and Translational Research Institute Dissemination and Implementation Science Center, University of California San Diego, La Jolla, CA USA; 4 Department of Psychiatry, University of California San Diego, La Jolla, CA USA; 5 Child and Adolescent Services Research Center, San Diego, CA USA; 6 The Global Action Research Center, San Diego, CA USA; 7 Department of Pediatrics, University of California San Diego Health, La Jolla, CA USA

**Keywords:** Community engagement, Capacity building, Policy advocacy, Underserved communities, COVID-19, Ethnographic methods, Mixed method

## Abstract

**Background:**

The COVID-19 pandemic exacerbated existing disparities in healthcare access and outcomes, particularly among underserved communities. As one site participating in the NIH-funded Community Engagement Alliance Against COVID-19, our focus was to address COVID-19 disparities by training immigrant and refugee communities to advocate for their needs by increasing capacity to campaign for policy-level changes.

**Objective:**

To evaluate the impact of a train-the-trainer policy advocacy program for ethnically-based community leaders within San Diego County using a mixed-methods evaluation.

**Methods:**

We partnered with a non-profit social change, intermediary organization to adapt a five-session, 4-hour per session training that was conducted over five weeks. A baseline survey, pre- and post-training surveys, and ethnographic documentation were employed during each session.

**Results:**

Among participants (*n* = 16), 50% were Latino(a), 25% were Somali Bantu, and 25% were Karen. Training results were relatively stable with slight variations in perceptions within and between sessions. The first session showed a slight decrease in confidence by the training participants, while sessions 3, 4, and 5 showed increases in confidence. Ethnographic documentation revealed that engagement patterns evolved over time, with the Latino(a) participants having the highest levels of engagement initially but with more equitable engagement across participants by the final session.

**Conclusion:**

These findings provide valuable feedback which will aid in the improvement of the training sessions for future use. This study also underscores the potential for community leaders to effectively advocate for policy changes and offers insights for future empowerment initiatives.

**Supplementary Information:**

The online version contains supplementary material available at 10.1186/s12889-024-20822-0.

## Background

Meaningful engagement of communities in research, public health, and policy change depends on the capacity of the community to engage in these activities [1, 2]. Community capacity can be advanced through targeted, hands-on trainings that provide an understanding of the specific process, as well as through sharing and teaching strategies that can be successfully used to partake in this process [3, 4]. Specifically, it is important to provide a clear understanding of how policy is created and implemented, and what steps community members can take to advocate for policy change locally, regionally, and nationally. Communities that have been historically marginalized can especially benefit from this type of training and guidance. The main principle underlying a capacity building lens to community engagement is the empowerment of the community to advocate for issues that are high priority for them. Research has indicated that achieving the greatest impact in dissemination and implementation efforts within historically underserved communities is closely linked to practicing cultural humility and sensitivity and collaborating with community organizations to bridge the gap between academia and communities [5, 6]. Furthermore, capacity-training for community leaders serves to enhance and refine their skills. The community members who participate in these trainings tend to overwhelmingly agree that the training has been beneficial to them and has enhanced their skillset in policy advocacy [7]. 

In our work with underserved communities in San Diego around COVID-19 preparedness and response, we identified key conditions that need to be met to achieve equitable COVID-19 testing and vaccine uptake. These were summarized in the form of theories of change and included several policy-relevant change recommendations [8]. The National Institutes of Health (NIH) initiated various rapid response funding opportunities for community-engaged research to address disparities in COVID-19 clinical trial participation, healthcare access, and vaccine uptake. One such NIH program is the Community Engagement Alliance Against COVID-19 Disparities (CEAL), which comprises community-academic partnerships in 11 states across the United States and concentrates on COVID-19 awareness and education research for the communities most impacted by the pandemic. The CEAL team in California includes an academic network involving 11 academic institutions, including the University of California San Diego. Previous work reporting results from the parent study include understanding attitudes and behaviors regarding COVID-19 vaccinations, with an emphasis on sources of trusted information, as well as descriptions of our multi-method ethnographic documentation of community engagement practices [8–10]. 

The objective of this study is to describe the development, delivery, and impact of a culturally and linguistically appropriate capacity-building training on policy advocacy designed for three underserved communities in San Diego County.

## Methods

### Study design

Share, Trust, Organize, Partner: The COVID-19 California Alliance (STOP COVID-19 CA) is an NIH-funded study part of the Community Engagement Alliance Against COVID-19 (CEAL) program with the goal of identifying and implementing strategies to address multi-level barriers regarding participation in COVID-19 research and programs for underserved communities. STOP COVID-19 CA includes 11 academic sites in California and over 70 community partner organizations. The UC San Diego site aimed to increase policy advocacy capacity within immigrant and refugee communities and track the impact of the training on policy advocacy outcomes and activities. This study uses a concurrent quantitative + qualitative mixed methods study design in which the qualitative data expanded and contextualized responses from the quantitative survey data.

### Development of the community advocacy training

Five in-person training sessions were refined by the Global Action Research Center (ARC) with input from the UCSD team. Session learning objectives and content were based on prior similar trainings led by the Global ARC for community partners. The content was adapted to the needs of the specific communities that were included in this training program. Appropriate language translations and live interpretation for monolingual speakers were provided to accommodate all participants. Training sessions were developed to last four hours each and included time at the beginning and end for evaluation activities.

### Community advocacy training

The training consisted of five four-hour sessions with distinct topics and learning objectives. The training plan and learning objectives for each session are outlined in Table 1 below.

**Table 1:** Sessions offered to community members and their learning objectives

Session and title	Learning objectives
Session 1: Orientation – Defining Values and Self-Interest	1. Have committed to completing the training (attend all sessions) 2. Be able to define their self-interest in their participation 3. Be able to identify the shared values within the group 4. Be able to identify the group’s interest
Session 2: Identifying and Building Power	1. Be able to define power within the context of community and public policy 2. Be able to articulate how power as defined in the session has shaped them and their environment 3. Be able to articulate the importance of and the power in building alliances with like-minded individuals and/or organizations
Session 3: Power Analysis	1. Be able to identify sectors within the community and assess each sector’s source and base of power as well as their self-interest 2. Be able to identify allies and opponents among the sectors in relation to the group’s concerns 3. Be able assess the power held by allies and opponents in relation to the group’s concerns
Session 4: Creating a Vision and Cutting an Issue	1. Have developed consensus on a common vision for what the group hopes to achieve with their campaign 2. Have operationally defined the components of their vision 3. Know how to identify an issue contained in the Vision that can become the focus of a campaign (based on the operational definitions) 4. Be able to apply the power analysis and scoping to their campaign
Session 5: Base Building	1. Have identified the people within their social/familial network 2. Have identified potential Weavers within their social/familial network 3. Be able to conduct a 1-on-1 interview 4. Have developed a message (hook) to attract people to the campaign 5. Have completed a broad campaign plan

Surveys were administered both before and after each training session with the purpose of capturing data at both time points. Surveys administered both pre- and post-session were designed to measure changes in perceived knowledge and confidence in achieving the learning objectives. The outline of survey administration is outlined below in Table 2, including a brief plan of ethnographic documentation.

**Table 2:** Outline of evaluation plan, timing of survey administration, and content evaluated

Measure	Timing	Session #	Content	Format
Baseline Profile	Pre	1	Experience organizing, age, ethnic group	Survey
Training Evaluation	Pre & Post	1 & 5	Attitude (importance), knowledge & ability	Survey
Learning Objectives	Pre & Post	2–5	Confidence based on learning objectives	Survey
Engagement	Post	2–5	Quantity & quality of engagement during each session	Survey
Ethnographic Documentation	During	1–5	Types and frequency of interactions, includes process data about # attendees	Observation

### Procedures

The physical location for the trainings was a Sudanese Community Center located in the “City Heights” neighborhood within San Diego County. Participants were enrolled and informed consent was obtained by research staff in person or by telephone/videoconference. Participants were provided a $100 USD gift card per training session, for up to five sessions and were asked to attend the session and complete all evaluation activities (Table 2).

### Debriefing session

All participants from the policy advocacy training were invited to a two-hour debriefing session led by the training facilitators and university evaluation team. The focus of this session was to share a summary of the quantitative evaluation data for the primary purpose of following up with members and ensuring that evaluation findings were well understood. In addition, participants were invited to share their ideas for applying the campaign training to their own work and community practice.

### Evaluation of the community advocacy training

A mixed-methods approach was used to evaluate the feasibility, acceptability, and impact of the advocacy training by participants. A combination of paper and online surveys, qualitative reflection activities, and ethnographic documentation were used. Table 2 provides an outline of the timing, content, and format of all evaluation measures. All paper surveys were entered by a research team member on Qualtrics for the purpose of consolidating data and data analysis.

### Session surveys

A separate training evaluation survey was administered at the beginning of the first training session and at the end of the fifth and final training. This survey was identical for both instances and was used to capture changes in information learned, perceptions, and ideas before and after the entire training. Learning objective surveys were administered at the beginning and end of each session, starting with session 2, and measured confidence based on the session-specific learning objectives.

All questions were asked using a Likert-type scale with and without a numerical scale, ranging from 1 to 5. The response options were presented with scaled text that ranged from either “Strongly disagree” to “Strongly agree” or “Not at all important” to “Very important”. For both types of questions, there was also an option of “Choose not to respond”. Session surveys also included open ended comment fields for elaborating on responses. For reference, see the supplementary file of this manuscript for the survey instruments of our baseline survey, training evaluation survey, and learning objectives survey (i.e. session surveys).

### Engagement survey

Individuals involved in each training session were asked to complete an “Engagement Survey’’ at the end of each training session. Individuals were asked to rate nine statements for both: “How well do the partners leading the workshop do each of the following?” and “How often do the partners leading the workshop do each of the following?”. The answer choices were either “Poor”, “Fair”, “Good”, “Very good”, “Excellent’’, or “Not applicable” or “Never”, “Rarely”, “Sometimes”, “Often”, “Always’’, or “Not applicable”, respectively.

The statements the individuals were asked included the following:

1. The focus is on needs important to the community.
2. All partners assist in establishing roles and related responsibilities for the partnership.
3. Community-engaged activities are continued until the goals (as agreed upon by all partners) are achieved.
4. The partnership adds value to the work of all partners.
5. The team builds on strength and resources within the community or patient population.
6. All partners’ ideas are treated with openness and respect.
7. All partners agree on the timeline for making shared decisions about the project.
8. The partnership’s processes support trust among all partners.
9. Mutual respect exists among all partners.

The results of these statements are shared in Supplemental Figs. 1 and 2. This survey was developed for a prior phase of this study and adapted for this phase [9, 10]. 

### Ethnographic documentation

Ethnographic methods were used to document the quality and degree of community member engagement within and across training sessions. Ethnographic documenters were part of the research team and were trained by experts in this type of observation and data collection; there was at least one documenter at each session. The documentation forms were adapted from a form previously used by the Global ARC and refined iteratively through pilot testing and debriefing meetings during an earlier linked study [9, 10]. The “Actors Form” documentation form allowed the research staff to gather information on various aspects of community members’ participation including attendance, time spent speaking, primary language used, whether an interpreter was used, arrival and departure time, and interruptions. Furthermore, we documented the type and content of interactions during the meeting. The types of interactions were predetermined prior to documenting and included “giving info”, “seeking info”, “agreement”, and “summation”. Each interaction was labeled as at least one of the four categories. For the purpose of the documentation, only interactions with the larger group were documented (i.e., interactions within the smaller groups were not captured for this description). Documenters also filled out a “Documenters Form” after the end of each training session they were present at, ensuring the reliability of the notes taken. The form captured the observations made by the documenters throughout each training session, encompassing various aspects such as the speaking time among different ethnic groups, the achievement of meeting objectives, and areas that may require further refinement or improvement such as language barriers and disparity in participation.

### Debriefing session

All participants from the policy advocacy training were invited to a two-hour debriefing session led by the training facilitators and university evaluation team. The focus of this session was to share a summary of the quantitative evaluation data for the primary purpose of following up with members and ensuring that findings were well understood. In addition, participants were invited to share their ideas for applying the campaign training to their own work and community practice. Community members who attended the debriefing session were compensated $100 USD. A total of 11 (68.8%) participants attended the in-person debriefing session that took place approximately four months following the last campaign training workshop.

### Population

The target population included adults aged 18 years or older who identified as a member of the Karen, Latino(a), and/or Somali Bantu communities in San Diego. Participants were identified through the collaboration of the Global ARC with community leaders and community organizers from these ethnically-based communities. The physical location for the trainings was a Sudanese Community Center located within the “City Heights” neighborhood in San Diego County. Participants were enrolled and informed consent was obtained by research staff in person or by telephone/videoconference.

### Analysis

Qualitative and quantitative data gathered from the trainings were analyzed using an explanatory mixed-methods approach, aiming to use qualitative data to explain our quantitative results. Quantitative and qualitative data were gathered from multiple documenters and compiled together for analysis throughout all five training sessions. Qualitative data from written, open-ended questions from the administered surveys and discussions captured by the ethnographic documenters were analyzed by using Microsoft Excel version 16.83 through a rapid thematic analysis. Themes were double coded by two research staff members and reviewed by the study team to resolve differences and agree on the final set of themes and coding process. Descriptive statistic techniques were used to analyze and convey the quantitative data gathered throughout the study period.

## Results

### Demographics

A total of 16 community leaders who reside in South San Diego participated. Of these, 10 were female (63%), 8 were Latino(a) (50%), 4 were Somali Bantu (25%), and 4 were Karen (25%). See Table 3 for participant demographics.

**Table 3:** Participant demographics (*n* = 16)

Variable	Category	Frequency	Percentage
Sex			
	Male	6	38%
	Female	10	63%
Ethnicity			
	Latino(a)	8	50%
	Somali Bantu	4	25%
	Karen	4	25%
Age			
	18–29	4	25%
	30–40	1	6%
	41–50	4	25%
	51–60	5	31%
	> 60	2	13%
Total:		16	100%

### Session results

The first set of the learning objective surveys, administered at the second training session, showed an average of a 0.2 decrease in points across the three questions asked. The remaining training session surveys administered on sessions 3, 4, and 5 saw an increase in average pre- and post-scores in every question. Session 3 saw an average increase, across all questions, of 0.5 points; Session 4 saw an average increase, across all questions, of 0.4 points; Session 5 saw an average increase, across all questions, of 0.4 points. See Table 4 for more details.

**Table 4:** Participant mean scores and mean change of scores per question, per session

Question	Mean Pre-score	Mean Post-score	Mean Change
Session 2: Identifying and Building Power			
I feel confident that I can define power within the context of community and public policy.	4.07	3.91	-0.16
I feel confident that I can articulate how power has shaped me and my environment.	4.00	3.82	-0.18
I feel confident that I can articulate the importance of and the power in building alliances with like-minded individuals and/or organizations.	4.15	4.00	-0.15
**Session 3: Power Analysis**			
I feel confident that I can identify sectors within my community and assess each sector’s source and base of power as well as their self-interest.	3.57	4.17	0.60
I feel confident that I can identify allies and opponents among the sectors in relation to the group’s concerns.	3.71	4.00	0.29
I feel confident that I can assess the power held by allies and opponents in relation to the group’s concerns.	3.57	4.15	0.58
**Session 4: Creating a Vision and Cutting an Issue**			
I feel confident that I can develop a consensus on a common vision for what the group hopes to achieve with their campaign.	3.80	4.07	0.27
I feel confident that I can operationally define the components of a group’s vision.	3.64	4.23	0.59
I feel confident that I can identify an issue that can become the focus of a campaign.	3.79	4.31	0.52
I feel confident that I can apply power analysis and scoping to my campaign.	4.00	4.39	0.39
**Session 5: Base Building**			
I feel confident that I can identify the people within my social/familial network.	4.09	4.25	0.16
I feel confident that I can identify potential Weavers within my social/familial network.	3.75	4.42	0.67
I feel confident that I can conduct a 1-on-1 interview.	3.83	4.25	0.42
I feel confident that I can develop a message (hook) to attract people to the campaign.	3.83	4.17	0.34
I feel confident that I can complete a broad campaign plan.	3.83	4.25	0.42

Table 5 reports the qualitative data that were collected from the first session. The first session has its own table due to the number of surveys administered on this date and the extensive discussion that took place. The table includes qualitative quotes collected from surveys completed on the first session where people provided open-ended comments about specific learning objectives, reasons for joining the training, and end of session reflections including: pre-session survey, post-session survey, pre-training survey, baseline survey, engagement survey, and the actors form completed by ethnographic documenters. A brief criterion of the thematic categories used in the analysis is found below:

#### Uncertainty

Participant conveyed some sort of uncertainty regarding the training session, learning objectives, or their own ability.

#### Praise

Participant conveyed praise for the study, study staff, or training session.

#### Suggestion

Participant conveyed a suggestion for the study, study staff, training session or to their peers (i.e., other participants).

#### Personal experience

Participant shared a personal experience or anecdote.

#### Barriers

Participant conveyed any sort of barrier to achieving the goal at hand including learning objectives and community advocacy and change.

#### Desire/Need for training

Participant conveyed a need for more training either due to eagerness or a perceived lack of skill.

**Table 5:** Session 1 qualitative data categorized by reoccurring themes among participants

Thematic category	Number of themes (%)		Example quotes		
Uncertainty	11 (20%)		“La verdad no sé y me gustaría aprender para poder abogar por mi comunidad.” “I really don’t know and I would like to learn to be able to advocate for my community.” (Latino(a) group) “I know very little about advocacy. I understand how it works but have never, personally, advocated for any policy.” (Karen group)		
Praise	6 (10.9%)		“I’m really happy to be here, since this is a really good opportunity. There are a lot of young people that care and use their voices and we need time and courage, but sometimes courage is hard to get. Once we develop and learn, I am really happy to have this opportunity because many times we don’t share our stories and listen to other people’s perspectives. And there are many things that I want to change but if we don’t speak no one will do it.” (Karen group) “Yo sí aprendí, lo que me quedó más en la mente es cómo se tiene que hacer una campaña efectiva. Si la comunidad no está presente en la toma de decisiones, no se puede cumplir, no se va hacer algo efectivo para el interés de la comunidad.” “I did learn and what stayed in my mind the most is how to make an effective campaign. If the community is not present in the decision-making process, it cannot be fulfilled, it is not going to be effective in the best interest of the community.” (Latino(a) group)		
Suggestion	9 (16.4%)		“If we want kids to feel safe we have to start with something small like cleaning parks, and this would make them more influential and they feel that they matter for the community and that would make them feel safe and to see change we have to start from something small and move to something bigger.” (Karen group) “…if we work together, we can achieve anything, we know our community needs us, but we need to make our voice heard. Try to engage with the community. You do need to speak English to do it. There are translators to help, but our voices matter, we all need to be involved.” (Somali group) “Me gustaría que se hagan más reuniones locales. Estoy para unir más a nuestra comunidad y otras minorías.” “I would like to see more local gatherings. I want to bring our community together, and other minorities closer together too.” (Latino(a) group)		
Personal experience	14 (25.5%)		“Por experiencia, no todas las agencias trabajan en equipo con otras, no tenemos mucho impacto las asociaciones pequeñas y tampoco fondos.” “From experience, not all agencies work as a team with others, small associations do not have much impact nor funds.” (Latino(a) group) “I have been engaged in advocacy at the local level but not much at the national level.” (Karen group)		
Barriers	5 (9.1%)		“Miro que ocupamos ayudar más a la comunidad latina. Ellos son ignorantes de muchas cosas en este país, especialmente la comunidad migrante.” “I see that we need to help the Latino community more. They are ignorant of many things in this country especially the migrant community.” (Latino(a) group) “Necesito más apoyo o entrenamiento de como hablar en público y como expresarme claramente y saber aprovechar los minutos que me otorguen.” “I need more support or training on how to speak in public and how to express myself clearly and know how to take advantage of the minutes that they give me.” (Latino(a) group)		
Desire/Need for training	10 (18.2%)		“I really don’t know and I would like to learn to be able to advocate for my community.” (Latino(a) group) “We need more union, information, participation in voting in participating in local politics in electing representatives.” (Karen group)		
Total:	55 (100%)				

Table 6 reports the qualitative data that were collected from the third, fourth, and fifth sessions. The quotes collected and included in the table are from surveys administered on these sessions including: Pre-session survey, post-session survey, engagement survey and the Actors form (completed by ethnographic documenters).

**Table 6:** Session 2, 3, 4, and 5 qualitative data categorized by recurring themes among participants

Thematic category	Number of themes (%)	Example quotes
Uncertainty	4 (9.5%)	“Yo sí entendí un poquito y hoy me perdí un poco; se me hizo que era demasiada información ya que no nos pones a investigar los otros tipos de posiciones en otras áreas pero sí creo que tengo un poco claro cómo identificar el problema y cómo empezar a trabajar y cómo aliarnos con otras personas para obtener su apoyo.” “I did understand a little bit and today I got a little bit lost and I thought it was a little bit too much information since you don’t put us to investigate the different types of positions in other areas but I think it’s a little bit clear on how to identify the problem and how to start working and how to learn with other people to get their support.” (Latino(a) group, session 4)
Praise	10 (23.8%)	“Well, after this training I have learned much more about campaigns than before and different structures of power. You have your own power. Like, I have certain ideas of who to look for or what kind of research I should do in order to get that needed attention. I like how you guys have been teaching us, like starting from the small little detail about the house. And then the power structure goes deeper to the powers of those who have the power. And we are from those types of things. I think it’s really helpful as an English learner. I think I learned much more than before, which I’m really grateful for. And thank you for sharing. Thank you.” (Karen group, session 3) “For me, it’s like every day I’m here, I learn something else. And it’s, it’s awesome how you guys are teaching us and how you’re gonna start getting here for most of us, and thank you for that.” (Latino(a) group, session 3)
Suggestion	12 (28.6%)	“Espero no contradecirme pero necesitamos establecer reglas de respeto cuando la gente habla, o no permitir el uso del cell solo en emergencia, la mayoría estuvimos comentando y no pusimos atención a lo que decían los demás y eso es falta de respeto.” “I hope I don’t contradict myself but we need to establish rules of respect when people speak, or not allow the use of the cell phone; only in an emergency, most of us were commenting and we did not pay attention to what others were saying and that is disrespectful.” (Latino(a) group, session 5)
Personal experience	9 (21.4%)	“Entiendo que es un proceso que a lo mejor cuesta mucho trabajo, hay veces que me molesto con (mi compañera) que me dice tranquila así es esto. Sí se que es el poder de la comunidad pero creo que necesitamos organizarnos como comunidad para que menos respeten, y no solo hay que que somos city heights o población de bajos recursos, y decir que somos de bajo recursos quiere decir que somos de mentalidad baja, salud, educación - todo es bajo para nosotras y se que el poder lo tenemos nosotros por eso tenemos que unirnos para no esperar 5 años y lograr cambio en menos tiempo.” “I understand that it is a process that may take a lot of work, there are times when I get upset with (my partner) who tells me to calm down, this is how it is. Yes, I know it is the power of the community, but I think we need to organize as a community so that they are less respected, and not only that we are city heights or a low-income population, and to say that we are low-income means that we are low-minded, health, education - everything is low for us and I know that we have the power, that is why we have to unite so as not to wait 5 years and achieve change in less time.” (Latino(a) group, session 3) “La falta de apoyo a las comunidades y familias de bajo recurso, ha marcado la falta de unión de grupos y organizaciones para que las autoridades nos tomen en cuenta, para abordar nuestras necesidades.” “The lack of support for low-income communities and families has marked the lack of union of groups and organizations so that the authorities take us into account, to address our needs.” (Latino(a) group, session 2)
Desire/Need for training	7 (16.7%)	“Hoy entendí el proceso y los niveles de poder, pero quiero aprender más.” “Today I understood the process and the power levels but want to learn more.” (Latino(a) group, session 2) “Felicidades, es de mucho valor este entrenamiento, me gustaría que hubiera seguimiento, u otros trainings que nos ayuden como asociación y comunidad.” “Congratulations, this training is very valuable, I would like there to be a follow-up, or other training that can help us as an association and community.” (Latino(a) group, session 4) “Podrían poner más ejemplos de los conocimientos teóricos y prácticos please?” “Could you give more examples of theoretical and practical knowledge please?” (Latino(a) group, session 5)
Total:	42 (100%)	

### Training evaluation

A separate survey was administered before and after completion of the 5th training session (i.e., at the beginning of session 1 and at the end of session 5). The average participant change score across all 4 questions was an increase of 0.03 points. When looking at specific questions, question 1 “How important is it for you to be engaged in local policy to advocate for your community’s needs and priorities?” had an average decrease of 0.17 points while question 3, “I understand how to engage in influencing local or national policy to reflect my community’s priorities.” had an average increase of 0.36. Questions 2 and 4 had smaller mean differences at -0.1 and + 0.02 respectively. See Table 7 for more details.

**Table 7:** Participant mean scores and mean change of scores pre- and post-training

Overall Training Evaluation			
Question	Mean Pre-score	Mean Post-score	Mean Change
How important is it for you to be engaged in local policy to advocate for your community’s needs and priorities?	4.77	4.60	-0.17
How important is it for you to be engaged in national policy to advocate for your community’s needs and priorities?	4.31	4.21	-0.10
I understand how to engage in influencing local or national policy to reflect my community’s priorities.	3.71	4.07	0.36
How skilled are you about advocating for local or national policy to reflect your community’s priorities?	3.21	3.23	0.02

The third question which had a notable average change in scores (+ 0.36) also included open-ended comments such as “Although I’m not proficient when it comes to creating changes in policy yet, I do know that in order to reflect the community’s priorities we must learn about the community itself.”, “Yes, I am learning, I have been participating in some changes and I know that the change does not depend on just one person but the entire community.” and “I have enough knowledge to know that alone, it is very difficult to achieve the goals and/or objectives.”

Lastly, there was an open-ended question at the end of this survey asking “How (if at all) did this training change your capacity to advocate for your community?”. Notable answers were “I learned how to run a campaign and now I know what steps to follow to be successful. I learned how to be an advocate and look at the interests of people in power, to see their own interest or that of the community”, “I am motivated to know more and take advantage of my professional experience to serve a greater purpose, to improve our mental health services.” and “It helped me understand where it all starts from, how it starts, and what to do when you start it.”

### Engagement

The majority of engagement principles received the highest rating of “excellent”. Principles 1 (“The focus is on needs important to the community”), 2 (“All partners assist in establishing roles and related responsibilities for the partnership”), and 3 (“Community-engaged activities are continued until the goals (as agreed upon by all partners) achieved”) were not perceived as positively as the others with “very good” being the response with the highest frequency. For how often the research team exhibits these principles, all nine had “Always”, the highest score, as the most frequent response item. See Supplemental Tables 1 and 2 for more details.

### Ethnographic evaluation

The amount of time (in seconds) that each participant spoke to the larger group was documented by ethnographic documenters, as well as the type of engagement. These counts are illustrated in Tables 8 and 9.

**Table 8:** Number of seconds spoken during large group discussions by session number and community group

Session	Latino(a) group	Karen group	Somali group
1	1883	1020	270
2	2749	600	0
3	1980	708	652
4	1310	648	349
5	840	120	120
Total:	8762	3096	1391

**Table 9:** Count of different instances of discussion by session number and community group

Session	Latino(a) group	Karen group	Somali group
1	8	6	7
2	15	5	4
3	15	3	3
4	21	0	6
5	5	1	4
Total:	64	15	24

*Note:* for each noted discussion, documenters could select more than one type of engagement, if applicable

During the training sessions, ethnographic documenters reported each instance of an individual volunteering to speak to the larger group. The amount of time and type of speech the participants engaged in was documented in all of the sessions. Table 8 shows the amount of time that participants spent talking to the larger group by session number. Sessions 2 and 3 had the highest combined total of seconds of group discussion at 3349 (55 min, 49 s) and 3340 (55 min, 40 s), respectively. Session 5 had the lowest number of combined seconds of group discussion. Note that this is not indicative of engagement since smaller group discussions were not considered for the part of the documentation. Table 8 shows the different types of engagement the documenters noted and the number of times they were noted across all five sessions. Out of the four types of speech, “giving information” had the highest frequency at 64 while summation only had six instances.

A common theme that arose among the first four sessions was regarding the difference in communication and engagement among the three community groups. Comments from documenters included “There was more engagement within small group and main group conversations from the Hispanic groups overall” (session 1), “the Latino organizations are significantly more vocal – even though they have more people, the proportion is still much higher” (session 2), “The Latino groups were more engaged compared to the Karen and Somali Bantu groups’’ (session 3), and “the Spanish-speakers participated much more than any other group” (session 4). However, it is notable to point out that in the fifth and last session a documenter pointed out that “This last session had a pretty equal variation in engagement across all groups. I think it might be because they got to know each other throughout the sessions and became comfortable with each other” (session 5). There were no disagreements or discrepancies between documenters regarding their comments and observations on engagement. Suggestions that the documenters offered were “to set up questions that require more direct communication between all of the groups”, “ having each person say their opinion to encourage them to talk. I suggest doing this in some exercises only since this would likely take up a lot of time”, “the interpreters often struggled to keep up with the presenters, and also translating for multiple people at once was difficult. In the future, I believe having more than one interpreter per language group would be helpful.” These suggestions were addressed by the training presenters and research staff by modifying the way in which questions were asked, by ensuring that there was an interpreter for each language at every session and by offering interpretation devices to participants to hear the presentation in their preferred language.

### Debriefing session

In the debriefing session, participants facilitated data interpretation and sense-making of the evaluation results, particularly the pattern of unchanged or slightly lower ratings of their knowledge of advocacy across sessions. To explain this, participants shared that because of the in-depth campaign development training, they discovered their personal knowledge gaps and recalibrated their ratings of knowledge more accurately by the end of their participation. Further, participants shared their ideas for capacity-building of campaign development and execution within their communities and across other local communities. In particular, participants expressed enthusiasm for collaborating across their community organizations to develop and execute a more unified campaign to address social determinants of health needs prioritized by the earlier workshops.

## Discussion

This study reports multi-method results from a five-day policy campaign training delivered to community leaders of Latino(a), Karen, and Somali Bantu communities. Overall, the quantitative data indicate that community members reported a small positive change in confidence across all of the sessions. Some questions, however, showed no change or slight negative effect. The qualitative data provided more context for the results.

The quantitative results presented in this study may be attributed to participants’ improved accuracy in self-assessment after engaging in the training sessions. It is plausible that, initially, participants had overestimated their knowledge and confidence in campaign planning. As the training progressed, they may have gained a more realistic perspective on their skills and areas that required further development. In a more general sense, this phenomenon, often known as the Dunning-Kruger effect, tells us that individuals tend to overestimate their knowledge of subjects [11]. While it is still possible that some community members may have worsened their skills, there is no qualitative evidence that may support this idea, which led our team to look at different explanations. Further work could emphasize qualitative methods more as a way to better capture how communities engage with and respond to the training program as cultural differences may affect how individuals respond to quantitative measures such as survey items.

There were notable discrepancies in talking time among the groups, with the Latino(a) group having a more significant proportion of participation, both anecdotally through ethnographic documentation and illustrated quantitatively in Table 8. This may reflect differences in communication styles or levels of comfort in participating within a larger group setting. Another factor is the Latino(a) groups likely included members who were previously known to each other while the other two groups did not. Further, some of the discrepancy can be attributed to the discrepant proportion of participants from distinct communities; half were part of Latino(a) organizations while the Karen and Somali Bantu groups each had a quarter of representation.

Despite these initial engagement imbalances, there was a shift across the sessions from more active participation by the Latino(a) groups to a more equitable engagement pattern across the three community groups. This change is also confirmed by the ethnographic documentation, which revealed a growing sense of collaboration and unity among participants throughout the training.

The debriefing session, conducted four months following the conclusion of the capacity training, proved to be a valuable experience for both the study staff and community members. It served as a platform for us to assess the program’s impact, allowing community members to voice their concerns and provide valuable feedback. Additionally, the session facilitated the ongoing development of a strong relationship between the research team and the community.

Furthermore, the debriefing session provided an opportunity for the research team to present the study’s findings, leading a discussion on their implications, identifying successful elements, acknowledging areas for improvement, and charting the course for future steps.

### Strengths and limitations

There are a few limitations to note. First, data collection was challenging due to the diverse languages spoken by participants and occasional recording issues. Also, as mentioned earlier, some groups seemed to be more eager to participate than other groups. These issues became less apparent throughout the sessions since we quickly addressed it by providing interpreters, radio devices, and adjusting the format of questions to facilitate participation. The sample size, while representative of the community leaders in South San Diego, may be considered relatively small for drawing generalizable conclusions. Furthermore, our sample is not fully representative of all ethnic minorities within our target population due to the population diversity of San Diego. Although the physical location of the training session was at the Sudanese Community Center, we did not include Sudanese community members in the training program and the choice of location was due to geographical convenience for the participating populations and space availability. Future exploration into ways to increase the reach of the training program would be beneficial.

The strengths of this study include the use of mixed methods, which allowed for a comprehensive understanding of the results by using qualitative data that provided context to the quantitative results. The study also demonstrated cultural sensitivity and awareness by providing translators and ensuring that the training was led by a trusted intermediary (the Global ARC), ensuring accessibility to a diverse group of participants. The ethnographic approach further enriched the study by capturing the engagement and communication in real-time.

Implications for future research and community empowerment are multiple. This study highlights the potential of community leaders to collaborate effectively in advocating for public health policy changes as well. Researchers and communities seeking to empower similar initiatives can draw from the lessons learned in this study, such as the value of culturally sensitive and appropriate training and the benefits of mixed methods research to gain a more comprehensive understanding of participants’ experiences and needs.

## Conclusion

In conclusion, this study engaged a diverse group of community leaders in a training program, revealing shifts in self-assessments, varying session results, and evolving engagement patterns. While initial confidence slightly decreased, later sessions demonstrated notable improvements. Engagement initially favored the Latino(a) group but equalized over time. A debriefing session held 4 months following the training sessions allowed for further community engagement and capacity building between the diverse groups of community members and the research team.

This study sheds light on the potential for community leaders to effectively advocate for public health policy changes through culturally sensitive training while offering valuable insights for future empowerment initiatives.

## Electronic supplementary material

Below is the link to the electronic supplementary material.

Supplementary Material 1

Supplementary Material 2

Supplementary Material 3

## Data Availability

The datasets generated and/or analyzed during the current study are not publicly available because of the small sample size and qualitative data collection, which would be identifying of participants. However, the co-author team will review data requests and that data will be made available as reasonably appropriate.
