# Supplementary material for: Increasing capacity for ethnically-based community leaders to engage in policy change: assessing the impact of a train-the-trainer approach
Source: BMC Public Health. 2025 Mar 11;25:968. doi: 10.1186/s12889-024-20822-0 (PMC11900054; doi:10.1186/s12889-024-20822-0)
Supplement: Supplementary file 1 — Supplementary Material 1 [file 12889_2024_20822_MOESM1_ESM.pdf]

STOP COVID-19 CA Community Advocacy Training Program

Pre-training Evaluation

1. How important is it for you to be engaged in local policy to advocate for your community's needs and priorities?
   - ☐ Not at all important
   - ☐ Low importance
   - ☐ Neutral
   - ☐ Important
   - ☐ Very important
   - ☐ Choose not to respond

   Please explain your answer:

2. How important is it for you to be engaged in national policy to advocate for your community's needs and priorities?
   - ☐ Not at all important
   - ☐ Low importance
   - ☐ Neutral
   - ☐ Important
   - ☐ Very important
   - ☐ Choose not to respond

   Please explain your answer:

3. I understand how to engage in influencing local or national policy to reflect my community's priorities.
   - ☐ Strongly disagree
   - ☐ Disagree
   - ☐ Neutral
   - ☐ Agree
   - ☐ Strongly agree
   - ☐ Choose not to respond

   Please explain your answer:

4. How skilled are you about advocating for local or national policy to reflect your community's priorities? *Circle the number that best corresponds to your answer choice with 1=not at all skilled and 5=very skilled.*

**Not at all skilled**				**Very skilled**	
1	2	3	4	5	Choose not to answer

Please explain your answer:

5. Do you have additional comments or would like to explain more?

Post-training Evaluation

1. How important is it for you to be engaged in local policy to advocate for your community's needs and priorities?

   - ☐ Not at all important
   - ☐ Low importance
   - ☐ Neutral
   - ☐ Important
   - ☐ Very important
   - ☐ Choose not to respond

Please explain your answer:

2. How important is it for you to be engaged in national policy to advocate for your community's needs and priorities?

   - ☐ Not at all important
   - ☐ Low importance
   - ☐ Neutral
   - ☐ Important
   - ☐ Very important
   - ☐ Choose not to respond

Please explain your answer:

3. I understand how to engage in influencing local or national policy to reflect my community's priorities.

   - ☐ Strongly disagree
   - ☐ Disagree
   - ☐ Neutral
   - ☐ Agree
   - ☐ Strongly agree
   - ☐ Choose not to respond

Please explain your answer:

4. How skilled are you about advocating for local or national policy to reflect your community’s priorities? *Circle the number that best corresponds to your answer choice with 1=not at all skilled and 5=very skilled.*

**Not at all skilled**				**Very skilled**	
1	2	3	4	5	Choose not to answer

Please explain your answer:

5. How (if at all) did this training change your capacity to advocate for your community?

6. Do you have additional comments or would like to explain more?

Baseline Profile Survey

1. How many years have you been an advocate for your community? _______________

2. What is your age (in years)? _______________

3. What is your gender?
   - ☐ Man
   - ☐ Woman
   - ☐ Transgender female or trans woman
   - ☐ Transgender male or trans man
   - ☐ Nonbinary, genderqueer, or genderfluid
   - ☐ I would describe my gender as: _______________
   - ☐ Prefer not to answer

4. Are you Hispanic or Latino?
   - ☐ No
   - ☐ Yes
   - ☐ Prefer not to answer

5. Which of the following best describes your race? *Please select all that apply.*
   - ☐ White
   - ☐ Black or African-American
   - ☐ Asian
   - ☐ American Indian or Alaska Native
   - ☐ Native Hawaiian or Other Pacific Islander
   - ☐ Prefer not to answer
   - ☐ Other: _______________________

6. Why did you join this training? ____

7. Please describe how you advocate for your community including impacting policy, if relevant.

Learning Objectives Survey (*pre-session and post-session*)

Session 2: Identifying and Building Power

1. I feel confident that I can define power within the context of community and public policy.
   - ☐ Strongly disagree
   - ☐ Disagree
   - ☐ Neutral
   - ☐ Agree
   - ☐ Strongly agree
   - ☐ Choose not to answer

Please explain your answer:

2. I feel confident that I can articulate how power has shaped me and my environment.
   - ☐ Strongly disagree
   - ☐ Disagree
   - ☐ Neutral
   - ☐ Agree
   - ☐ Strongly agree
   - ☐ Choose not to answer

Please explain your answer:

3. I feel confident that I can articulate the importance of and the power in building alliances with likeminded individuals and/or organizations.
   - ☐ Strongly disagree
   - ☐ Disagree
   - ☐ Neutral
   - ☐ Agree
   - ☐ Strongly agree
   - ☐ Choose not to answer

Please explain your answer:

Session 3: Power Analysis

1. I feel confident that I can identify sectors within my community and assess each sectors source and base of power as well as their self-interest.

   - ☐ Strongly disagree
   - ☐ Disagree
   - ☐ Neutral
   - ☐ Agree
   - ☐ Strongly agree
   - ☐ Choose not to answer

Please explain your answer:

2. I feel confident that I can identify allies and opponents among the sectors in relation to the group's concerns.

   - ☐ Strongly disagree
   - ☐ Disagree
   - ☐ Neutral
   - ☐ Agree
   - ☐ Strongly agree
   - ☐ Choose not to answer

Please explain your answer:

3. I feel confident that I can assess the power held by allies and opponents in relation to the group's concerns.

   - ☐ Strongly disagree
   - ☐ Disagree
   - ☐ Neutral
   - ☐ Agree
   - ☐ Strongly agree
   - ☐ Choose not to answer

Please explain your answer:

Session 4: Creating a Vision and Cutting an Issue

1. I feel confident that I can develop consensus on a common vision for what the group hopes to achieve with their campaign.

   - ☐ Strongly disagree
   - ☐ Disagree
   - ☐ Neutral
   - ☐ Agree
   - ☐ Strongly agree
   - ☐ Choose not to answer

Please explain your answer:

2. I feel confident that I can operationally define the components of a group's vision.

   - ☐ Strongly disagree
   - ☐ Disagree
   - ☐ Neutral
   - ☐ Agree
   - ☐ Strongly agree
   - ☐ Choose not to answer

Please explain your answer:

3. I feel confident that I can identify an issue that can become the focus of a campaign.

   - ☐ Strongly disagree
   - ☐ Disagree
   - ☐ Neutral
   - ☐ Agree
   - ☐ Strongly agree
   - ☐ Choose not to answer

Please explain your answer:

4. I feel confident that I can apply power analysis and scoping to my campaign.

   - ☐ Strongly disagree
   - ☐ Disagree
   - ☐ Neutral
   - ☐ Agree
   - ☐ Strongly agree
   - ☐ Choose not to answer

Please explain your answer:

Session 5: Base Building

1. I feel confident that I can identify the people within my social/familial network.

- ☐ Strongly disagree
- ☐ Disagree
- ☐ Neutral
- ☐ Agree
- ☐ Strongly agree
- ☐ Choose not to answer

Please explain your answer:

2. I feel confident that I can identify potential Weavers within my social/familial network.

- ☐ Strongly disagree
- ☐ Disagree
- ☐ Neutral
- ☐ Agree
- ☐ Strongly agree
- ☐ Choose not to answer

Please explain your answer:

3. I feel confident that I can conduct a 1-on-1 interview.

- ☐ Strongly disagree
- ☐ Disagree
- ☐ Neutral
- ☐ Agree
- ☐ Strongly agree
- ☐ Choose not to answer

Please explain your answer:

4. I feel confident that I can develop a message (hook) to attract people to the campaign.

- ☐ Strongly disagree
- ☐ Disagree
- ☐ Neutral
- ☐ Agree
- ☐ Strongly agree
- ☐ Choose not to answer

Please explain your answer:

5. I feel confident that I can complete a broad campaign plan.

- ☐ Strongly disagree
- ☐ Disagree
- ☐ Neutral
- ☐ Agree
- ☐ Strongly agree
- ☐ Choose not to answer

Please explain your answer:

Evaluación Previa a la Capacitación

1. ¿Qué tan importante es para usted participar en la política local para abogar por las necesidades y prioridades de su comunidad?

   ☐ Nada importante
   ☐ De poca importancia
   ☐ Neutral
   ☐ Importante
   ☐ Muy importante
   ☐ Prefiero no contestar

Por favor explique su respuesta:

2. ¿Qué tan importante es para usted participar en la política nacional para abogar por las necesidades y prioridades de su comunidad?

   ☐ Nada importante
   ☐ De poca importancia
   ☐ Neutral
   ☐ Importante
   ☐ Muy importante
   ☐ Prefiero no contestar

Por favor explique su respuesta:

3. Sé cómo participar para influir en la política local o nacional para reflejar las prioridades de mi comunidad.

   ☐ Totalmente en desacuerdo
   ☐ En desacuerdo
   ☐ Neutral
   ☐ De acuerdo
   ☐ Totalmente de acuerdo
   ☐ Prefiero no contestar

Por favor explique su respuesta:

4. ¿Qué tan hábil es usted para abogar por una política local o nacional que refleje las prioridades de su comunidad? *Encierre en un círculo el número que mejor corresponda a su opción de respuesta con 1=nada hábil y 5 =muy hábil*.

**Sin habilidad**				**Con mucha habilidad**	
1	2	3	4	5	Prefiero no contestar

Por favor explique su respuesta:

5. ¿Tiene algún comentario adicional o le gustaría agregar algo más?

Evaluación Posterior a la Capacitación

1. ¿Qué tan importante es para usted participar en la política local para abogar por las necesidades y prioridades de su comunidad?

   - ☐ Nada importante
   - ☐ De poca importancia
   - ☐ Neutral
   - ☐ Importante
   - ☐ Muy importante
   - ☐ Prefiero no contestar

Por favor explique su respuesta:

2. ¿Qué tan importante es para usted participar en la política nacional para abogar por las necesidades y prioridades de su comunidad?

   - ☐ Nada importante
   - ☐ De poca importancia
   - ☐ Neutral
   - ☐ Importante
   - ☐ Muy importante
   - ☐ Prefiero no contestar

Por favor explique su respuesta:

3. Sé cómo participar para influir en la política local o nacional para reflejar las prioridades de mi comunidad.

   - ☐ Totalmente en desacuerdo
   - ☐ En desacuerdo
   - ☐ Neutral
   - ☐ De acuerdo
   - ☐ Totalmente de acuerdo
   - ☐ Prefiero no contestar

Por favor explique su respuesta:

4. ¿Qué tan hábil es usted para abogar por una política local o nacional que refleje las prioridades de su comunidad? *Encierre en un círculo el número que mejor corresponda a su opción de respuesta con 1=nada hábil y 5 =muy hábil*.

**Sin habilidad**				**Con mucha habilidad**	
1	2	3	4	5	Prefiero no contestar

Por favor explique su respuesta:

5. ¿Cómo (si es que cambió) esta capacitación cambió su capacidad para abogar por su comunidad?

6. ¿Tiene algún comentario adicional o le gustaría agregar algo más?

Encuesta de Perfil de Referencia

Nombre: ________________________

Grupo comunitario: ______________________________

¿Cuales son los últimos 4 dígitos de su número de teléfono celular? ___________

1. ¿Cuántos años ha estado abogando por su comunidad? _______________

2. ¿Cuál es su edad? _____ años

3. ¿Cuál es su género?
   - ☐ Hombre
   - ☐ Mujer
   - ☐ Mujer transgénero o mujer trans
   - ☐ Hombre transgénero o hombre trans
   - ☐ No binario, genderqueer o género de no binaridad (genderfluid)
   - ☐ Describo mi género como: _______________
   - ☐ Prefiero no contestar

4. ¿Es usted hispano o latino?
   - ☐ No
   - ☐ Sí
   - ☐ Prefiero no contestar

5. ¿Cuál de las siguientes describe mejor su raza? *Por favor seleccione todas las que correspondan.*
   - ☐ Caucásica
   - ☐ Negra o afroamericana
   - ☐ Asiática
   - ☐ India americana o nativa de Alaska
   - ☐ Nativa hawaiana u otra isleña del Pacífico
   - ☐ Prefiero no contestar
   - ☐ Otra: _______________________

6. ¿Por qué está tomando esta capacitación?

   ____

7. Por favor describa cómo aboga por su comunidad, incluida la política de impacto (si aplica)

   ____

Encuesta de Objetivos de Aprendizaje (antes y después de la sesión)

Sesión 2: Identificando y Construyendo Poder

1. Estoy seguro(a) que puedo definir el poder dentro del contexto de la comunidad y las políticas públicas.

- ☐ Totalmente en desacuerdo
- ☐ En desacuerdo
- ☐ Neutral
- ☐ De acuerdo
- ☐ Totalmente de acuerdo
- ☐ Prefiero no contestar

Por favor explique su respuesta:

2. Estoy seguro(a) de que puedo explicar cómo el poder me ha marcado a mí y a mi entorno.

- ☐ Totalmente en desacuerdo
- ☐ En desacuerdo
- ☐ Neutral
- ☐ De acuerdo
- ☐ Totalmente de acuerdo
- ☐ Prefiero no contestar

Por favor explique su respuesta:

3. Estoy seguro(a) de que puedo explicar la importancia y el poder de construir alianzas con personas u organizaciones de ideas afines.

- ☐ Totalmente en desacuerdo
- ☐ En desacuerdo
- ☐ Neutral
- ☐ De acuerdo
- ☐ Totalmente de acuerdo
- ☐ Prefiero no contestar

Por favor explique su respuesta:

Sesión 3: Análisis de Poder

1. Estoy seguro(a) que puedo identificar sectores dentro de mi comunidad y evaluar la fuente y la base de poder de cada sector, así como su interés propio.

   ☐ Totalmente en desacuerdo
   ☐ En desacuerdo
   ☐ Neutral
   ☐ De acuerdo
   ☐ Totalmente de acuerdo
   ☐ Prefiero no contestar

Por favor explique su respuesta:

2. Estoy seguro(a) de que puedo identificar aliados y opositores entre los sectores en relación con las preocupaciones del grupo.

   ☐ Totalmente en desacuerdo
   ☐ En desacuerdo
   ☐ Neutral
   ☐ De acuerdo
   ☐ Totalmente de acuerdo
   ☐ Prefiero no contestar

Por favor explique su respuesta:

3. Estoy seguro(a) de que puedo evaluar el poder que tienen los aliados y los oponentes en relación con las preocupaciones del grupo.

   ☐ Totalmente en desacuerdo
   ☐ En desacuerdo
   ☐ Neutral
   ☐ De acuerdo
   ☐ Totalmente de acuerdo
   ☐ Prefiero no contestar

Por favor explique su respuesta:

Sesión 4: Crear una Visión y Evitar un Problema

1. Estoy seguro(a) que puedo llegar a un consenso sobre una visión común de lo que el grupo espera lograr con su campaña.

   ☐ Totalmente en desacuerdo
   ☐ En desacuerdo
   ☐ Neutral
   ☐ De acuerdo
   ☐ Totalmente de acuerdo
   ☐ Prefiero no contestar

Por favor explique su respuesta:

2. Estoy seguro(a) de que puedo definir operativamente los componentes de la visión de un grupo.

   ☐ Totalmente en desacuerdo
   ☐ En desacuerdo
   ☐ Neutral
   ☐ De acuerdo
   ☐ Totalmente de acuerdo
   ☐ Prefiero no contestar

Por favor explique su respuesta:

3. Estoy seguro(a) que puedo identificar un tema que puede convertirse en el enfoque de una campaña.

   ☐ Totalmente en desacuerdo
   ☐ En desacuerdo
   ☐ Neutral
   ☐ De acuerdo
   ☐ Totalmente de acuerdo
   ☐ Prefiero no contestar

Por favor explique su respuesta:

4. Estoy seguro(a) de que puedo aplicar el análisis de poder y el alcance a mi campaña.

   ☐ Totalmente en desacuerdo
   ☐ En desacuerdo
   ☐ Neutral
   ☐ De acuerdo
   ☐ Totalmente de acuerdo
   ☐ Prefiero no contestar

Por favor explique su respuesta:

Sesión 5: Construcción de Fundamentos

1. Estoy seguro(a) de que puedo identificar a las personas dentro de mi red familiar/social.

   ☐ Totalmente en desacuerdo
   ☐ En desacuerdo
   ☐ Neutral
   ☐ De acuerdo
   ☐ Totalmente de acuerdo
   ☐ Prefiero no contestar

Por favor explique su respuesta:

2. Estoy seguro(a) de que puedo identificar a los Tejedores *[o* Weavers*, que son quienes saben tender puentes entre los diferentes sectores de la sociedad]* potenciales dentro de mi red familiar/social.

   ☐ Totalmente en desacuerdo
   ☐ En desacuerdo
   ☐ Neutral
   ☐ De acuerdo
   ☐ Totalmente de acuerdo
   ☐ Prefiero no contestar

Por favor explique su respuesta:

3. Estoy seguro(a) de que puedo realizar una entrevista cara a cara.

   ☐ Totalmente en desacuerdo
   ☐ En desacuerdo
   ☐ Neutral
   ☐ De acuerdo
   ☐ Totalmente de acuerdo
   ☐ Prefiero no contestar

Por favor explique su respuesta:

4. Estoy seguro(a) de que puedo desarrollar un mensaje (de enganche) para atraer personas a la campaña.

   ☐ Totalmente en desacuerdo
   ☐ En desacuerdo
   ☐ Neutral
   ☐ De acuerdo
   ☐ Totalmente de acuerdo
   ☐ Prefiero no contestar

Por favor explique su respuesta:

5. Estoy seguro(a) de que puedo realizar un amplio plan de campaña.

- ☐ Totalmente en desacuerdo
- ☐ En desacuerdo
- ☐ Neutral
- ☐ De acuerdo
- ☐ Totalmente de acuerdo
- ☐ Prefiero no contestar

Por favor explique su respuesta:
