# Supplementary figures and images for: Increasing capacity for ethnically-based community leaders to engage in policy change: assessing the impact of a train-the-trainer approach
Source: BMC Public Health. 2025 Mar 11;25:968. doi: 10.1186/s12889-024-20822-0 (PMC11900054; doi:10.1186/s12889-024-20822-0)

Supplemental Figure 1. Quality of engagement results by statement and frequency


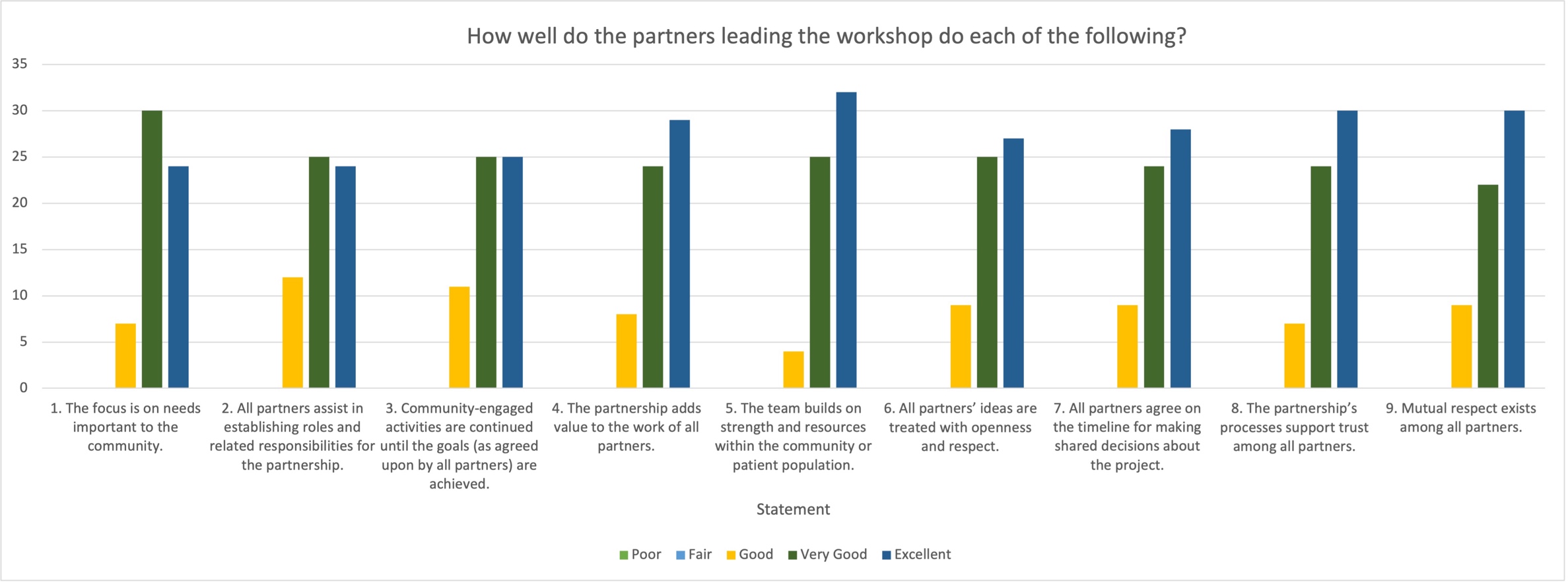

Supplement: Supplementary file 2 — Supplementary Material 2 [file 12889_2024_20822_MOESM2_ESM.docx]

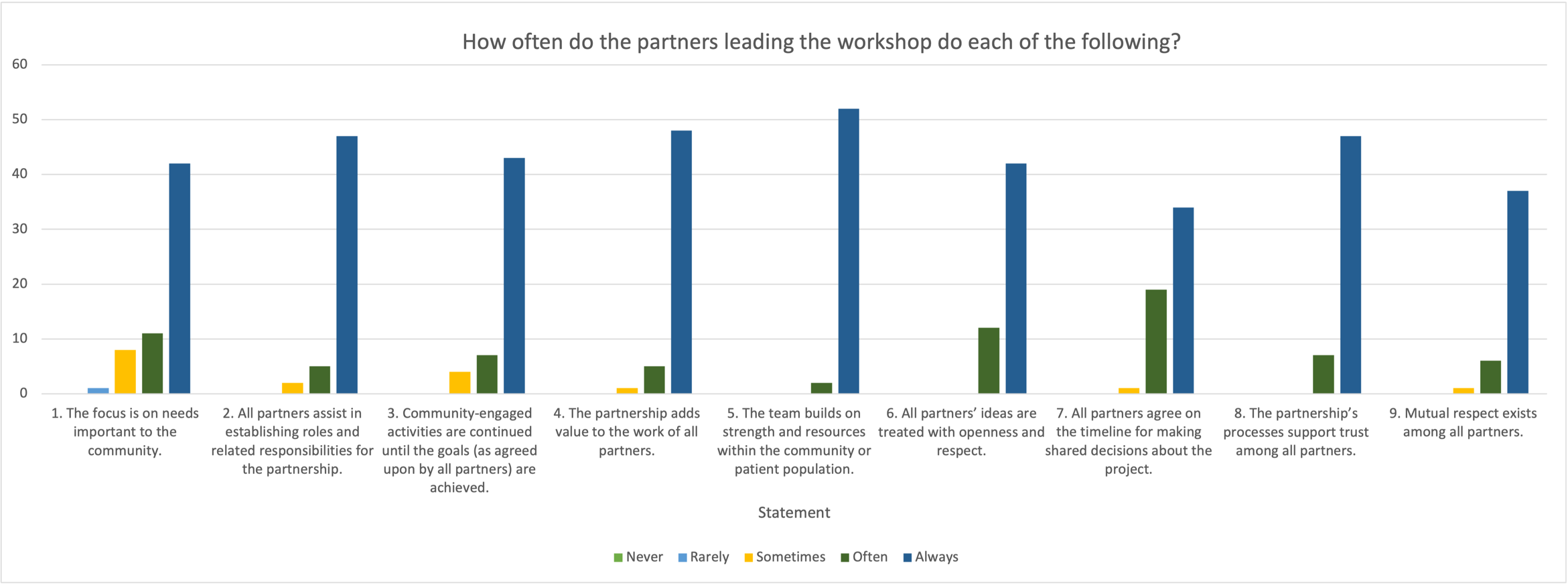
Supplemental Figure 2. Frequency of engagement results by statement and frequency

Supplement: Supplementary file 3 — Supplementary Material 3 [file 12889_2024_20822_MOESM3_ESM.docx]
